# A giant renal angiomyolipoma (AML) in a patient with septo-optic dysplasia (SOD)

**DOI:** 10.1186/s40001-014-0046-8

**Published:** 2014-09-09

**Authors:** Marcin Cichocki, Marek Sosnowski, Zbigniew Jablonowski

**Affiliations:** Clinic of Urology at Medical University, Military Medical Academy University Teaching Hospital-Central Veterans Hospital, Żeromskiego 113, 90-549 Lodz, Poland

**Keywords:** Angiomyolipoma, Hypopituitarism, Septo-optic dysplasia

## Abstract

Angiomyolipoma (AML) is a rare benign renal tumor occurring in about 0.3 to 3% of the general population. Most frequently it takes the form of small single tumors occurring sporadically or accompanying tuberous sclerosis (Bourneville-Pringle disease). In some cases the tumor may reach a very large size and be a cause of various serious complications. This case description concerns a 26-year-old female patient, suffering from hypopituitarism, hypothyroidism and binocular blindness during the course of septo-optic dysplasia, in whom a giant, left renal AML was diagnosed and treated surgically. According to the authors’ knowledge this was the first reported case of a huge size AML in a patient with de Morsier syndrome.

## Background

Angiomyolipoma (AML) is a rare benign renal tumor histologically containing elements of smooth muscle, adipose tissue and blood vessels in various proportions. It occurs in 0.3 to 3% of the general population, four-fold more frequently in women and the peak of incidence falls between the 30th and 50th years of life [1]. Most frequently (80%) these are small, singular tumors which occur unilaterally and occasionally. In the remaining cases they coexist with tuberous sclerosis (Bourneville-Pringle disease) and they may be bilateral and multiple and achieve significant sizes [2,3]. A tumor which is bigger than 10 cm and affects a whole organ is considered a ‘giant’ AML [4]. Septo-optic dysplasia (de Morsier syndrome) is a very rare syndrome of congenital defects consisting of optic nerve hypoplasia, hypopituitarism, and agenesis of the septum pellucidum [5,6].

## Case presentation

**Figure 1:**
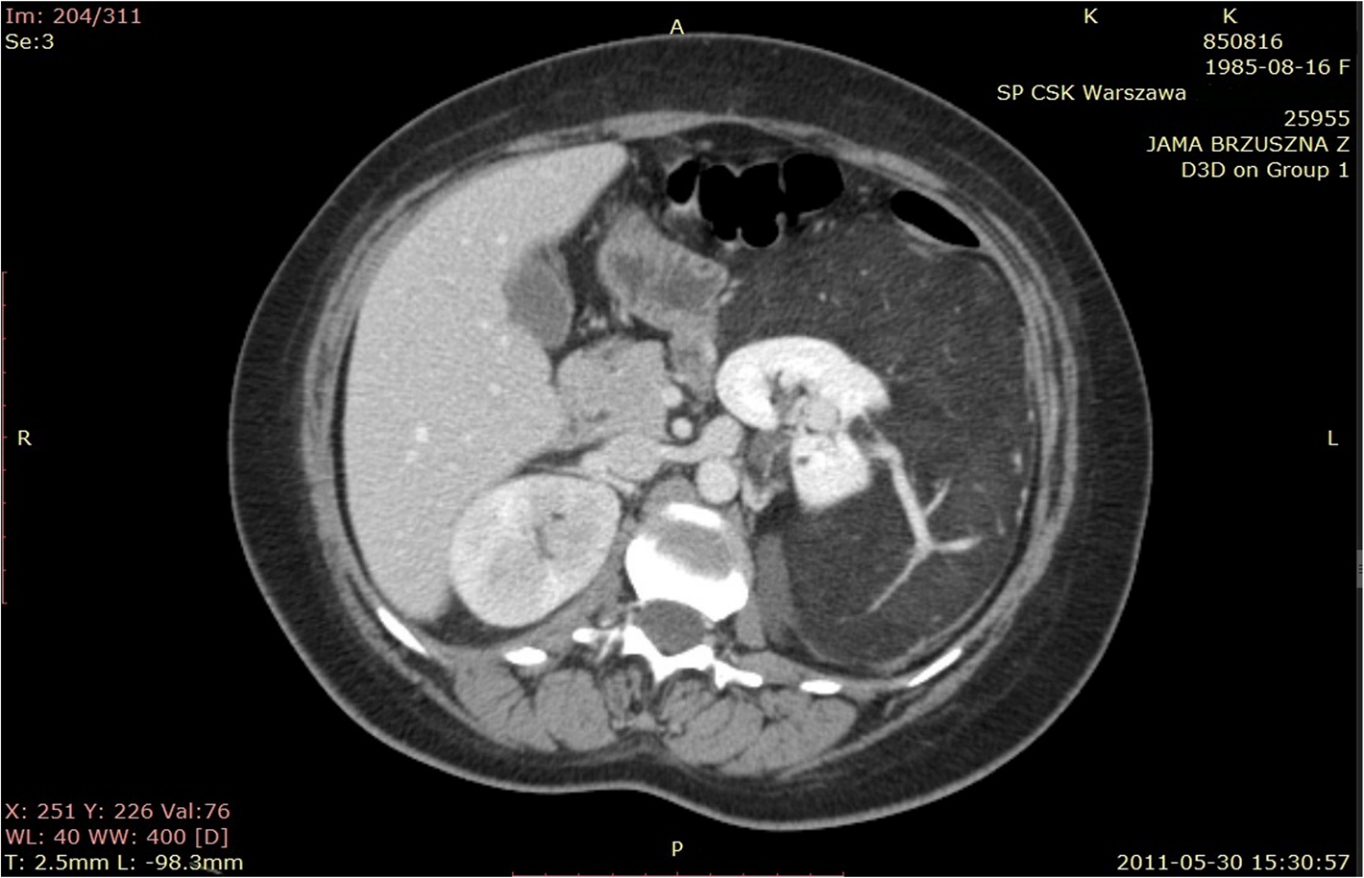
**Computer tomography (CT) angiography - visible tumor and pathological vessel.**

**Figure 2:**
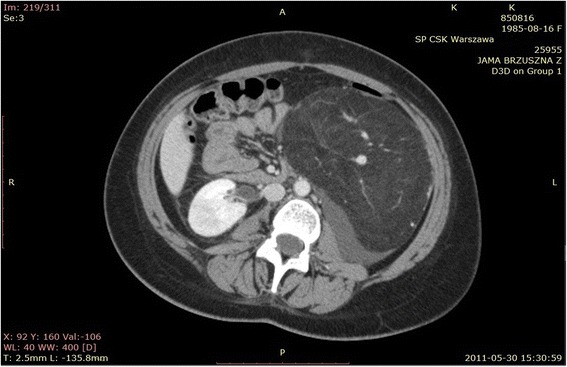
**A different scan with a visible tumor.**

**Figure 3:**
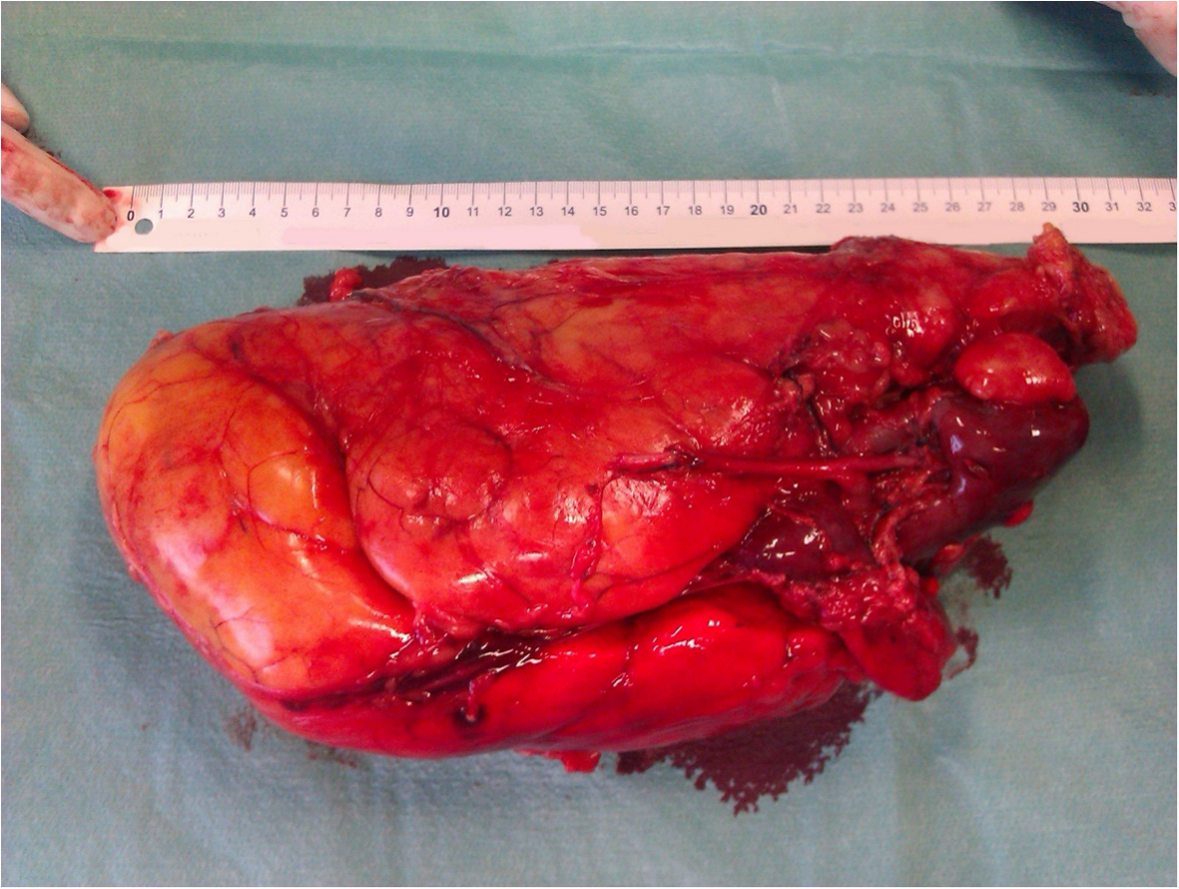
**A post-surgical specimen with a visible fragment of ureter and healthy renal parenchyma.**

**Figure 4:**
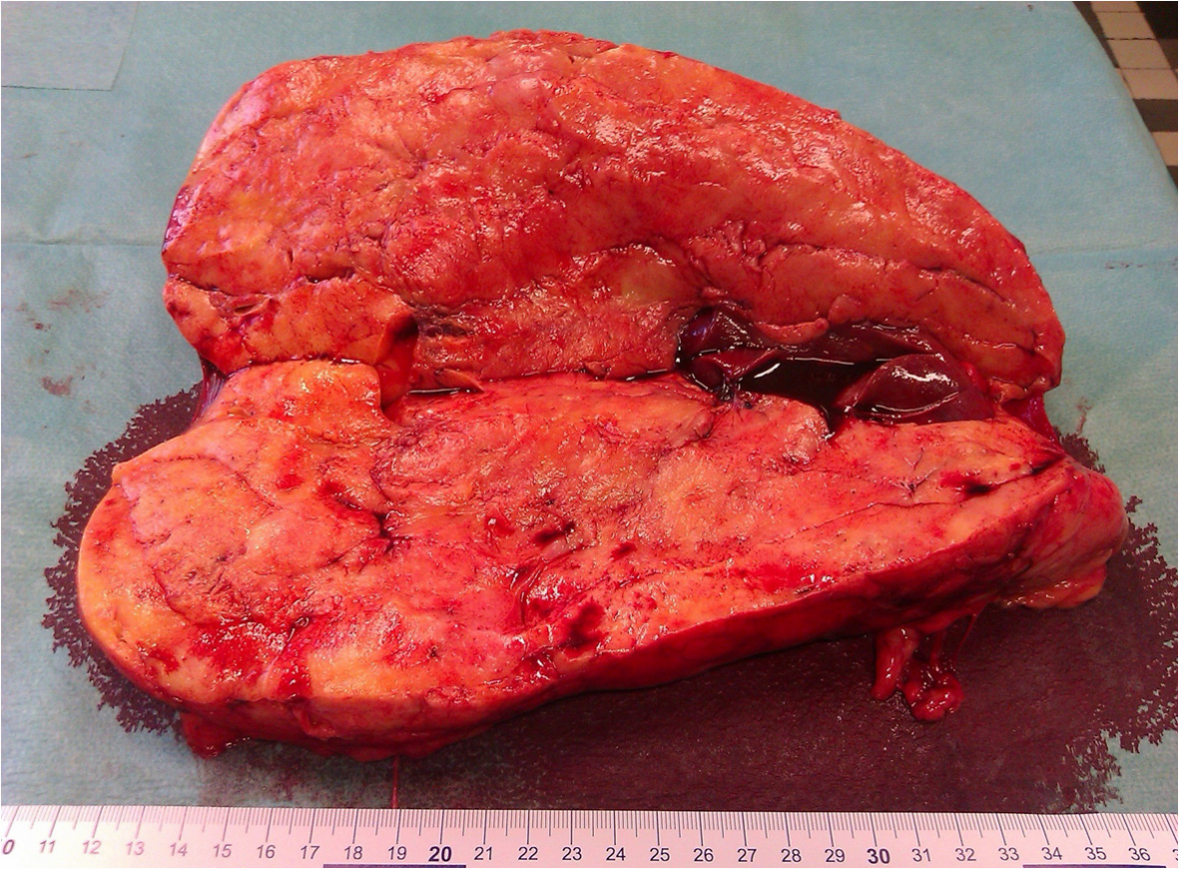
**A post-surgical specimen with a visible fragment of healthy renal parenchyma.**

The patient, a 26-year-old woman with a diagnosis of hypopituitarism, hypothyroidism and binocular blindness in the course of septo-ocular dysplasia was admitted to the Clinic of Urology because of a large tumor of the left kidney. The patient was 139 cm tall, weighed 52 kg and her BMI amounted to 27 kg/m^2^. Her blood pressure was 120/70 mmHg, heart rate 70/minute, creatinine levels 66 μmol/l, and her electrolytes, blood count and urinalysis were normal. The lesion had been diagnosed incidentally in another medical centre during investigation of persistent stomach ache with a suspicion of pyelonephritis. During case examination a perceptible mobile pathological resistance was reported on the left side of the stomach from the costal arch up to the iliac fossa. On abdominal ultrasonography (USG) examination, a large, intra-abdominal and left flank heterogeneous tumor with dimensions 170 × 78 mm, mostly hyperechogenic (consisting probably of adipose tissue with little vascularization observed in Doppler color) and probably coming out of the left kidney, was reported. The right kidney was 108 mm long, with no urinary retention and no deposits. The diagnostics were extended by computer tomography (CT) angiography of the abdomen with contrast, in which the following was stated: numerous AMLs in the left kidney, the biggest coming out from its lower part and affecting the whole paranephric space and going down to the level of iliac fossa with dimensions of about 226 × 95 × 104 mm. Except for this the other intra-abdominal organs revealed no abnormalities (Figures 1, 2). The patient qualified for surgical treatment. The whole kidney with the entire tumor was removed (Figure 3 and Figure 4) by performing a median transperitoneal incision. Due to the risk of serious bleeding, the priority was to reach the renal vessels. The procedure was performed without any complications with the loss of 100 ml of blood. Further recovery proceeded without any complications. Histopathological examination confirmed the diagnosis of AML.

## Discussion

AML is one of the rarely occurring solid tumors of the kidney (2 to 6%). Most frequently they are diagnosed by chance during imaging examinations and they do not reach large sizes. They occur multifocally in about 1/3 and bilaterally in about 15% of all cases. They coexist with tuberous sclerosis (Bourneville-Pringle disease) with a frequency of about 20%. In such patients AML is diagnosed in 40 to 80% with a 2 to 3% risk of coexistence of renal cell carcinoma (RCC). AML has a mild nature and it does not produce metastases. Most frequently it proceeds asymptomatically. It may induce hematuria and uncharacteristic pain [1-3]. The most serious and dangerous complication for health from AML is spontaneous bleeding into the retroperitoneal space from pathological vessels, referred to as Wünderlich syndrome [7]. Diagnosis is based mainly on imaging examinations: USG and CT (presence of adipose tissue within the tumor mass of density of 10 Hounsfield units is a characteristic feature), magnetic resonance imaging (MRI), angiography. Tumors larger than 4 cm are usually symptomatic and these are the tumors which require control every six months [8]. The incidence of AML larger than 10 cm in individuals without a diagnosis of tuberous sclerosis is extremely rare. The commonly applied methods of treatment include: cryoablation, embolization, tumorectomy, partial resection of the kidney, or nephrectomy. The choice and scope of treatment is determined by: tumor size, its vascularization, symptoms and comorbidities [9]. Septo-optic dysplasia (de Morsier syndrome) is a syndrome of congenital defects which occurs at a frequency of 1:50,000 and which consists of: optic nerve hypoplasia, hypopituitarism, and agenesis of the septum pellucidum. Its etiology is multivariate and still not fully known. Recent reports claim a possible role of the *HESX1* gene which is responsible for the development of the pituitary and central parts of the brain. Boys fall ill from this syndrome just as often as girls [10]. So far, an increased frequency of coexistence of renal carcinomas, including AML, has not been described. Herein, we have presented the first case of a large AML in a patient with de Morsier syndrome.

## Conclusions

We have presented an extremely rare case of the coexistence of congenital defects in the form of septo-optic dysplasia associated with a giant renal AML. The patient required a precise diagnostic work-up and radical surgical treatment.

## Consent

Written informed consent was obtained from the patient for publication of this case report and any accompanying images. A copy of the written consent is available for review by the Editor-in-Chief of this journal.

## Abbreviations

AML: angiomyolipoma
BMI: body mass index
CT: computer tomography
MRI: magnetic resonance imaging
RCC: renal cell carcinoma
SOD: septo-optic dysplasia
USG: ultrasonography
